# Neuroprotective Impact of Linagliptin against Cadmium-Induced Cognitive Impairment and Neuropathological Aberrations: Targeting SIRT1/Nrf2 Axis, Apoptosis, and Autophagy

**DOI:** 10.3390/ph16081065

**Published:** 2023-07-27

**Authors:** Hany H. Arab, Ahmed H. Eid, Shuruq E. Alsufyani, Ahmed M. Ashour, Azza A. K. El-Sheikh, Hany W. Darwish, Gehan S. Georgy

**Affiliations:** 1 Department of Pharmacology and Toxicology, College of Pharmacy, Taif University, P.O. Box 11099, Taif 21944, Saudi Arabia; s.alsofyani@tu.edu.sa; 2 Department of Biochemistry, Faculty of Pharmacy, Cairo University, Cairo 11562, Egypt; 3 Department of Pharmacology, Egyptian Drug Authority (EDA)—Formerly NODCAR, Giza 12654, Egypt; drahmedhamdy2007@yahoo.com (A.H.E.); gehan_gorgy11@hotmail.com (G.S.G.); 4 Department of Pharmacology and Toxicology, College of Pharmacy, Umm Al Qura University, P.O. Box 13578, Makkah 21955, Saudi Arabia; amashour@uqu.edu.sa; 5 Basic Health Sciences Department, College of Medicine, Princess Nourah bint Abdulrahman University, P.O. Box 84428, Riyadh 11671, Saudi Arabia; aaelsheikh@pnu.edu.sa; 6 Department of Pharmaceutical Chemistry, College of Pharmacy, King Saud University, P.O. Box 11451, Riyadh 11451, Saudi Arabia; hdarwish@ksu.edu.sa

**Keywords:** linagliptin, cadmium, autophagy, apoptosis, SIRT1

## Abstract

Cadmium is an environmental contaminant associated with marked neurotoxicity and cognitive impairment. Linagliptin, a dipeptidyl peptidase-4 (DPP-4) inhibitor, has demonstrated promising neuroprotection against cerebral ischemia and diabetic dementia. However, there has been no study of its effect on cadmium-induced cognitive deficits. In the present work, linagliptin’s prospective neuroprotective effects against cadmium-evoked cognitive decline were examined in vivo in rats. The molecular pathways related to oxidative stress, apoptosis, and autophagy were investigated. Histology, immunohistochemistry, ELISA, and biochemical assays were performed on brain hippocampi after receiving linagliptin (5 mg/kg/day). The current findings revealed that cadmium-induced learning and memory impairment were improved by linagliptin as seen in the Morris water maze, Y-maze, and novel object recognition test. Moreover, linagliptin lowered hippocampal neurodegeneration as seen in histopathology. At the molecular level, linagliptin curtailed hippocampal DPP-4 and augmented GLP-1 levels, triggering dampening of the hippocampal neurotoxic signals Aβ42 and p-tau in rats. Meanwhile, it enhanced hippocampal acetylcholine and GABA and diminished the glutamate spike. The behavioral recovery was associated with dampening of the hippocampal pro-oxidant response alongside SIRT1/Nrf2/HO-1 axis stimulation. Meanwhile, linagliptin counteracted hippocampal apoptosis markers and inhibited the pro-apoptotic kinase GSK-3β. In tandem, linagliptin activated hippocampal autophagy by lowering SQSTM-1/p62 accumulation, upregulating Beclin 1, and stimulating AMPK/mTOR pathway. In conclusion, linagliptin’s antioxidant, antiapoptotic, and pro-autophagic properties advocated its promising neuroprotective impact. Thus, linagliptin may serve as a management approach against cadmium-induced cognitive deficits.

## 1. Introduction

Several body systems are negatively affected by cadmium, a heavy metal with a potent toxicity prospect. The widespread use of cadmium in agriculture, industry, and anthropogenic activities makes its exposure unavoidable. In the human body, cadmium has a long biological half-life with an average of 14 years owing to its low rate of excretion from the body. Hence, cadmium triggers marked toxicity to different body organs [1]. Ample evidence exists that cadmium prompts evident toxicity to the nervous system with several manifestations, including olfactory dysfunction, memory impairment, and Alzheimer-like symptoms. Notably, epidemiological reports have characterized that repeated exposure to cadmium is associated with diminished cognition in human subjects [2]. Yet, the exact molecular mechanisms that underlie cadmium’s neurotoxic effects are still unresolved.

Several mechanisms have been suggested for explaining cadmium-induced neurotoxicity, including neuronal oxidative aberrations and apoptotic cell death, particularly in the hippocampus, the main part of the brain that controls memory acquisition and learning ability [3,4,5]. Excessive reactive oxygen species (ROS) generation prompts multiple redox perturbations, including lipid peroxidation. Meanwhile, disturbance of the hippocampal nuclear-factor-erythroid-2-related factor-2/heme oxygenase-1 (Nrf2/HO-1) cascade and depletion of multiple antioxidant moieties have been characterized as crucial factors in cadmium neurotoxicity [3,4]. Virtually, several antioxidant genes including glutathione peroxidase (GPx) and HO-1 are controlled by Nrf2, a redox-sensitive transcription factor. In cadmium-triggered neurotoxicity, inhibition [3] as well as activation [5] of Nrf2/HO-1 pathway have been reported. Hence, further exploration of the role of Nrf2 is warranted in cadmium-induced neurotoxicity in vivo. In the same regard, silent-information-regulated transcription factor 1 (SIRT1) protein depletion has previously been shown in mice with cadmium-induced memory deficits [4]. By resisting oxidative stress and apoptosis, SIRT1, a cytoprotective NAD^+^-dependent deacetylase, has been reported to protect memory and enhance learning in rodents [6]. When neurons are exposed to exaggerated oxidative stress, the mitochondrial apoptotic pathway prevails as the main programmed cell death pathway. During this process, the B-cell lymphoma-2 protein (Bcl-2) is downregulated while the Bcl-2-associated x protein (Bax) is overexpressed [4,5,7]. Notably, apoptosis of hippocampal neurons adversely affects memory acquisition and learning in animals [4].

During exposure to stressors, including cadmium, autophagy has been proposed as a protective mechanism that promotes cell survival [8]. As an evolutionarily conserved catabolic process, autophagy rids the cells of damaged cellular organelles such as mitochondria and misfolded macromolecules. In the context of cadmium-induced neurotoxicity, conflicting data have been described where activation [9] as well as inhibition [8,10,11] of autophagy have been reported in neuronal cells in vitro. Moreover, the in vivo effects of cadmium on the animal brain have not been sufficiently described [5]. Relevant to autophagy regulation, a significant role of the 5′adenosine-monophosphate-activated protein kinase/ mammalian target of rapamycin (AMPK/mTOR) cascade has been identified by several studies. In the autophagy process, AMPK/mTOR cascade is activated [9] for clearing neurons of diverse neurotoxic signals [12].

Growing evidence has revealed that repeated cadmium exposure is associated with multiple Alzheimer (AD)-like manifestations including memory deficits and impaired learning ability in vivo [3,4]. At the molecular level, deposition of the amyloid β-peptide (Aβ) protein aggregates prevails in the brain as extracellular plaques. Intracellularly, deposition of phospho-tau (p-tau) in the form of neurofibrillary tangles has also been described in cadmium-induced cognitive dysfunction in rodents [12]. These plaques trigger neuritic dystrophy, synaptic damage, and neuronal loss, particularly in the hippocampus region of the brain, culminating in cognitive and memory dysfunction [1].

**Figure 1:**
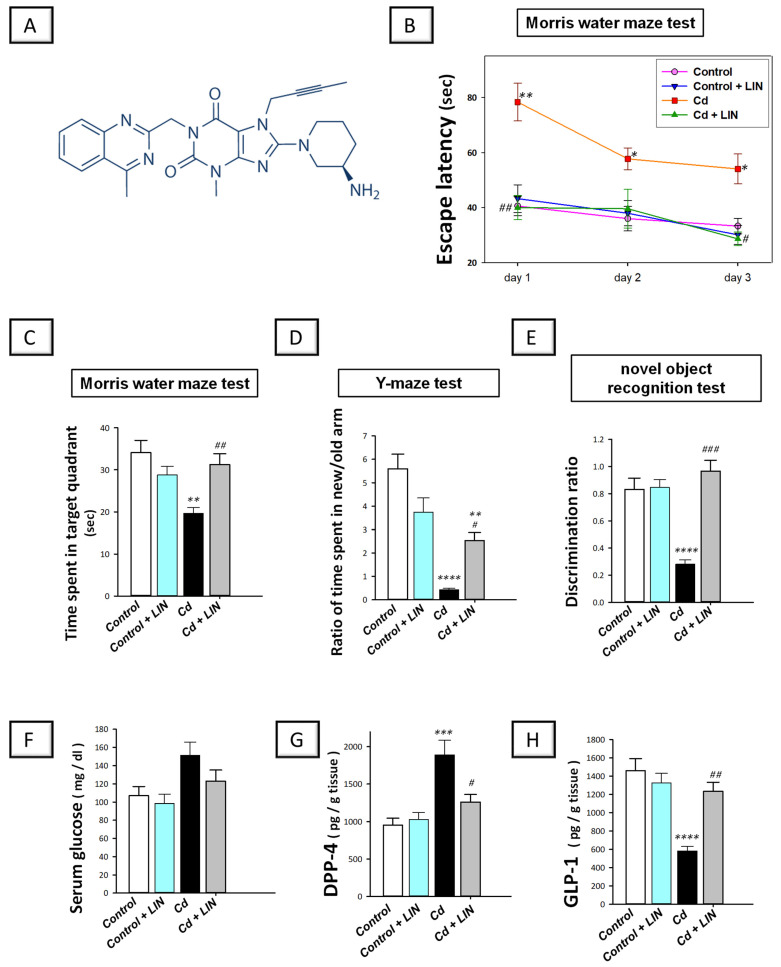
Linagliptin ameliorates cadmium-induced retention and recognition memory impairment in rats. (**A**) Linagliptin chemical structure. The behavioral study was carried out using the Morris water maze (MWM) paradigm, Y-maze test, and novel object recognition test. (**B**) During the course of training in the MWM, linagliptin reduced the time needed for animals to reach the submerged platform (escape latency). (**C**) In the probe test of the MWM, linagliptin improved the retention memory in rats by significantly increasing the time spent in the target quadrant after the removal of the submerged platform. (**D**) The Y-maze test was used to examine the short-term recognition memory after 1 h of the training session. Herein, linagliptin improved the short-term working memory of rats by increasing the ratio of the time spent in the new/old arm. (**E**) The novel object recognition test was implemented after 24 h of the training session for the examination of the long-term recognition memory. Herein, linagliptin improved the long-term recognition memory of rats by increasing the discrimination ratio. Relevant to linagliptin’s mode of action, the levels of serum glucose and hippocampal DPP-4 and GLP-1 were studied. Linagliptin elicited no significant effect on serum glucose levels in normoglycemic rats (**F**) and lowered hippocampal DPP-4 (**G**) with an elevation in hippocampal GLP-1 (**H**) in rats. *n* = 6 in each group (mean ± standard error of the mean). A *p*-value of less than 0.05 was significant. * p < 0.05, ** p < 0.01, *** p < 0.001, or **** p < 0.0001, compared to control; *^#^ p* < 0.05, *^##^ p* < 0.01, *or ^###^ p* < 0.001, compared to cadmium (Tukey’s test for multi-comparisons and one-way ANOVA). Cd, cadmium chloride; DPP-4, dipeptidyl peptidase-4; GLP-1, Glucagon-like peptide-1; LIN, linagliptin.

Linagliptin is an FDA-approved selective dipeptidyl peptidase-4 (DPP-4) inhibitor (chemical structure is illustrated in Figure 1A). Classically, the anti-diabetic drug linagliptin is used to treat type 2 diabetes [13,14]. Moreover, it has demonstrated several beneficial actions beyond its anti-diabetic ability including the amelioration of inflammatory bowel disease [15], chronic kidney damage [16], and testicular injury [17] in normoglycemic animals without prompting hypoglycemia. Consistently, studies in the clinical setting have revealed that linagliptin administration is associated with a low risk of hypoglycemia [18,19,20]. Regarding the link between DPP-4 inhibition and potential neuroprotection, the incretin hormone glucagon-like peptide-1 (GLP-1) is produced by intestinal L-cells, and it serves as a neurotransmitter in brain cells [21]. Evidence exists that linagliptin exerts neuroprotection mainly by DPP-4 inhibition, thereby increasing the half-life of GLP-1 incretin, favoring its spike in the brain of rodents [21]. In the context of linagliptin’s neuroprotective potential, in vitro studies have revealed that linagliptin dampens Aβ42-triggered neurotoxicity in neuronal cells in vitro [22]. In agreement, in vivo reports have revealed marked neuroprotective features of linagliptin against the cognitive decline observed in murine models of streptozotocin-induced diabetic dementia [13], cerebral ischemia in type 2 diabetes [18], and high-fat-evoked cognitive deficit in PS19 transgenic mice [19]. However, there has been no research on whether linagliptin might improve the cognitive decline caused by cadmium in rats. In the current study, linagliptin, a selective DPP-4 inhibitor with marked antioxidant [13,14,18,23] and anti-apoptotic [24,25] features, was assessed for its potential neuroprotective effects against cadmium-evoked cognitive decline. As part of our study, we looked at the potential mechanisms that are involved in neuronal oxidative aberrations, apoptosis, and autophagy, especially the cytoprotective pathways of SIRT1/Nrf2 and AMPK/mTOR. Additionally, the behavioral outcomes and several neurotoxic signals were explored. Notably, normoglycemic animals were used in the current set of experiments to dissect the potential neuroprotective impact of linagliptin away from its glucose-lowering ability. This is especially noteworthy because hyperglycemia has been linked to worsening neuronal degeneration [26]. Virtually, several studies have described the use of normoglycemic animals for investigating the beneficial effects of linagliptin in rodent models of inflammatory bowel disease [15], chronic kidney damage [16], and testicular injury [17].

## 2. Results

### 2.1. Linagliptin Improves Cadmium-Evoked Retention and Recognition Memory Impairment in Rats

In vivo, rats were tested on spatial learning and memory retention using the Morris water maze (MWM) paradigm [27]. During the training days in the MWM, cadmium chloride led to a significant increase in the time needed for rats to reach the submerged platform (escape latency), declaring impaired spatial learning ability (Figure 1B). Compared to the cadmium-intoxicated group, linagliptin significantly diminished the escape latency of animals on day 1 (*p* < 0.01) and day 3 of the training (*p* < 0.05). One day after the final trial, the hidden platform was removed to assess memory retention in animals. In the probe test, a significant difference existed among the experimental groups in the time spent in the target quadrant (F (3, 20) = 7.443, *p* = 0.0015), as illustrated in Figure 1C. Cadmium chloride led to a significant decline (*p* < 0.01) in the time spent in the target quadrant by 42.4%, revealing dysfunctional retention memory. Compared to the cadmium-intoxicated group, the animal’s retention memory improved in response to linagliptin, as demonstrated by a significantly prolonged (*p* < 0.01) time in the target quadrant by 58.94%. Of note, there were no significant changes in the retention memory when linagliptin was administered only to control rats.

We also examined the ability of linagliptin to ameliorate the short-term recognition memory impairment in rats using the Y-maze test that was performed 1 h after the training session. Herein, a significant difference existed among the experimental groups in the ratio of the time spent in the new/old arm (F (3, 20) = 21.24, *p* < 0.0001), as illustrated in Figure 1D. Cadmium chloride led to a significant lowering (*p* < 0.0001) of the ratio of the time spent in the new/old arm by 92.4%, revealing impaired recognition memory in the short term. Compared to the cadmium-intoxicated group, animal recognition memory improved in response to linagliptin, as demonstrated by a significant increase (*p* < 0.0001) in this ratio. Of note, there were no significant changes in the short-term recognition memory test when linagliptin was administered only to control rats. We further assessed the ability of linagliptin to rescue recognition memory deficit in rats in the long term (24 h after the training session). In this regard, the novel object recognition test (NORT) assesses the spontaneous tendency of animals to explore novel objects [28]. Herein, a significant difference existed among the experimental groups in the discrimination ratio (F (3, 20) = 21.22, *p* < 0.0001), as illustrated in Figure 1E. Cadmium chloride led to a significant lowering (*p* < 0.0001) in the discrimination ratio by 66.1%, demonstrating an impaired recognition memory in the long term. Compared to the cadmium-intoxicated group, animal recognition memory was improved in response to linagliptin, as demonstrated by a significant increase (*p* < 0.001) in the discrimination ratio. Of note, there were no significant changes in the long-term recognition memory test when linagliptin was administered only to control rats. Together, the present findings characterize the efficacy of linagliptin to improve dysfunctional spatial learning and memory impairment in response to cadmium intoxication in rats. Moreover, linagliptin rescued the cadmium-induced recognition memory deficit in rats in the short and long term.

Relevant to its mode of action, the effects of linagliptin on the serum glucose levels of normoglycemic rats were investigated. There was a non-significant change in serum glucose levels among all experimental groups (Figure 1F). Regarding hippocampal dipeptidyl peptidase-4 (DPP-4) and glucagon-like peptide-1 (GLP-1) in rats (Figure 1G,H), significant differences existed among the experimental groups in the levels of DPP-4 (F (3, 20) = 11.13, *p* = 0.0002) and GLP-1 (F (3, 20) = 14.55, *p* < 0.0001). Cadmium chloride led to a significant increase (*p* < 0.001) in DPP-4 content by 98.3% together with a significant (*p* < 0.0001) decline in GLP-1 by 60.1%. Compared to the cadmium-intoxicated group, these changes were counteracted in response to linagliptin, as demonstrated by a significant lowering (*p* < 0.05) in DPP-4 content by 33.5% alongside a significant elevation (*p* < 0.01) in GLP-1 by 111.9%. In the previous parameters, there were no significant changes when linagliptin was administered only to control rats. Overall, besides its ability to dampen DPP-4 and increase hippocampal GLP-1, the current findings reveal that linagliptin does not interfere with serum glucose levels in euglycemic rats.

### 2.2. Linagliptin Attenuates the Degenerative Histological Changes Induced by Cadmium in the Hippocampi of Rats

**Figure 2:**
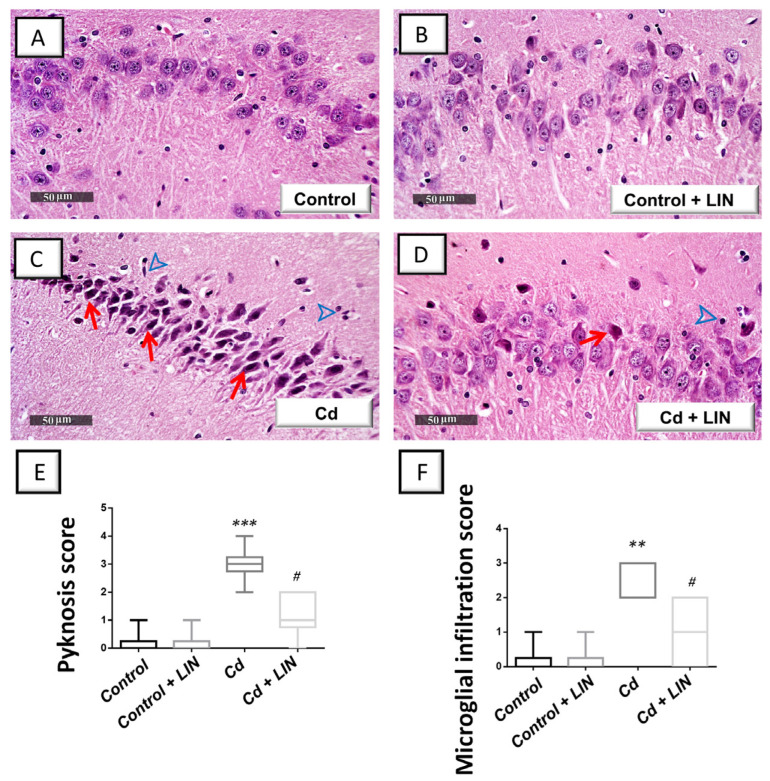
Linagliptin mitigates cadmium-induced neuronal degeneration and glial cell infiltration in the hippocampi of rats. Hippocampal sagittal sections were inspected by a light microscope after staining with hematoxylin and eosin (H-E). An intact structure of the hippocampus and organized distribution of the hippocampal layers were seen in the control (**A**) and control + linagliptin groups (**B**). Moreover, the hippocampus area of both groups revealed intact pyramidal neurons with distinct nuclear and subcellular details. (**C**) The hippocampus area of Cd group showed marked degeneration, neuronal loss, pyknotic pyramidal neurons with invisible subcellular details (red arrow), and reactive microglial cell infiltrates (arrowhead). (**D**) Cd + linagliptin group demonstrated attenuation of the hippocampal pathological changes and demonstrated few records of neuronal degenerative changes (red arrow). However, some reactive glial cell infiltrates were still seen (arrowhead). (**E**,**F**) The scores of pyknosis and microglial cell infiltration were significantly lowered by linagliptin administration to cadmium-intoxicated animals. *N* = 6 in each group (median with interquartile range). A *p*-value of less than 0.05 was significant. ** p < 0.01, or *** p < 0.001, compared to control; *^#^ p* < 0.05, compared to cadmium (Dunn’s test for multi-comparisons and Kruskal–Wallis test). Cd, cadmium chloride; LIN, linagliptin.

To further characterize the favorable effects of linagliptin, the morphological changes evoked by cadmium were explored in the hippocampi by microscopy. The histological images from the control (Figure 2A) and control + linagliptin (Figure 2B) revealed intact architecture of the hippocampus with well-organized hippocampal layers, and intact pyramidal neurons demonstrating distinct nuclear and subcellular details. The hippocampus of the Cd group (Figure 2C) showed severe degenerative changes and neuronal loss with abundant records of pyknotic pyramidal neurons alongside invisible subcellular details. Notably, moderate edema in the brain matrix and considerable reactive microglial cell infiltrates were observed. Linagliptin administration to cadmium-intoxicated animals (Figure 2D) counteracted the histomorphological changes detected in the hippocampus area and revealed a few records of neuronal degenerative changes and an improved picture of intact neurons with distinct subcellular details and an intact brain matrix. Yet, persistent reactive glial cell infiltrates were noted. These data suggest that linagliptin mitigated the cadmium-induced histopathological changes and hippocampal neuronal degeneration in rats. To further characterize the hippocampal histopathological damage in animals, quantification of the neuropathological damage was applied as the scores of pyknosis and microglial cell infiltration (Figure 2E,F). Herein, a significant difference existed among the experimental groups in pyknosis scores (H (3, 20) = 18.03, *p* = 0.0004) and microglial cell infiltration scores (H (3, 20) = 16.57, *p* = 0.0009). In this context, the scores of pyknosis (*p* < 0.001) and microglial cell infiltration (*p* < 0.01) were significantly increased in cadmium-intoxicated animals. In response to linagliptin administration to cadmium-intoxicated animals, the pyknosis (*p* < 0.05) and microglial cell invitation (*p* < 0.05) scores were significantly attenuated by 61.1% and 62.5%, respectively.

### 2.3. Linagliptin Lowers the Expression of the Neurotoxic Aβ42 and p-Tau in the Hippocampi of Cadmium-Intoxicated Rats

**Figure 3:**
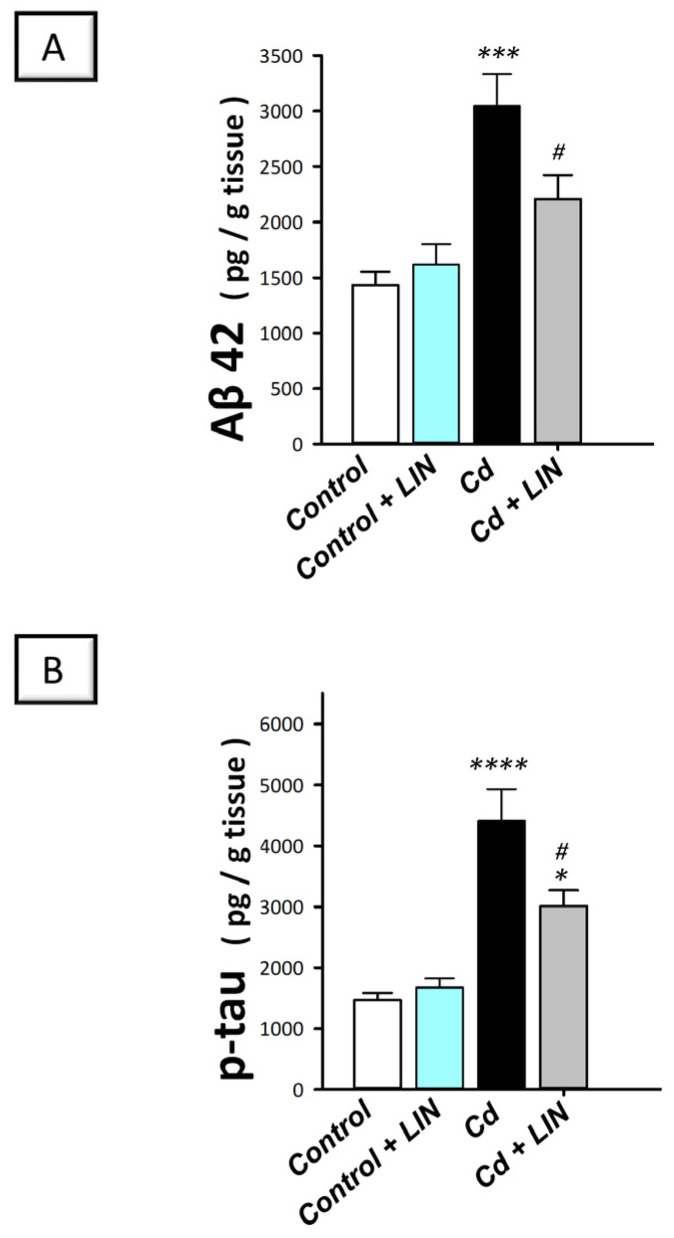
Linagliptin lowers hippocampal amyloid beta 42 (Aβ42; (**A**)) and phosphorylated tau (p-tau; (**B**)) in cadmium-intoxicated rats. *N* = 6 in each group (mean ± standard error of the mean). A *p*-value of less than 0.05 was significant. * p < 0.05, *** p < 0.001, or **** p < 0.0001, compared to control; *^#^ p* < 0.05, compared to cadmium (Tukey’s test for multi-comparisons and one-way ANOVA). Cd, cadmium chloride; LIN, linagliptin.

To characterize the underlying molecular events linked to the pathogenesis of cadmium-induced cognitive decline and Alzheimer-like molecular aberrations, we investigated the protein expression of the neurotoxic Aβ42 and p-tau, hallmark signals of Alzheimer’s pathology [1,12]. Herein, a significant difference existed among the experimental groups in the protein expression of Aβ42 (F (3, 20) = 11.92, *p* = 0.0001) and p-tau (F (3, 20) = 19.49, *p* < 0.0001), as illustrated in Figure 3. Cadmium chloride led to a significant increase in Aβ42 (*p* < 0.001) by 112.4% (Figure 3A) and p-tau (*p* < 0.0001) by 200.4% (Figure 3B). Compared to the cadmium-intoxicated group, these neurotoxic signals were reversed in response to linagliptin, as demonstrated by a significant downregulation of Aβ42 (*p* < 0.05) and p-tau protein expression (*p* < 0.05) by 27.4% and 31.7%, respectively. Of note, there were no significant alterations in these neurotoxic markers when linagliptin was administered only to control rats.

### 2.4. Linagliptin Augments Acetylcholine and GABA Neurotransmitters and Suppresses Glutamate and Acetylcholine Esterase in the Hippocampi of Cadmium-Intoxicated Rats

**Figure 4:**
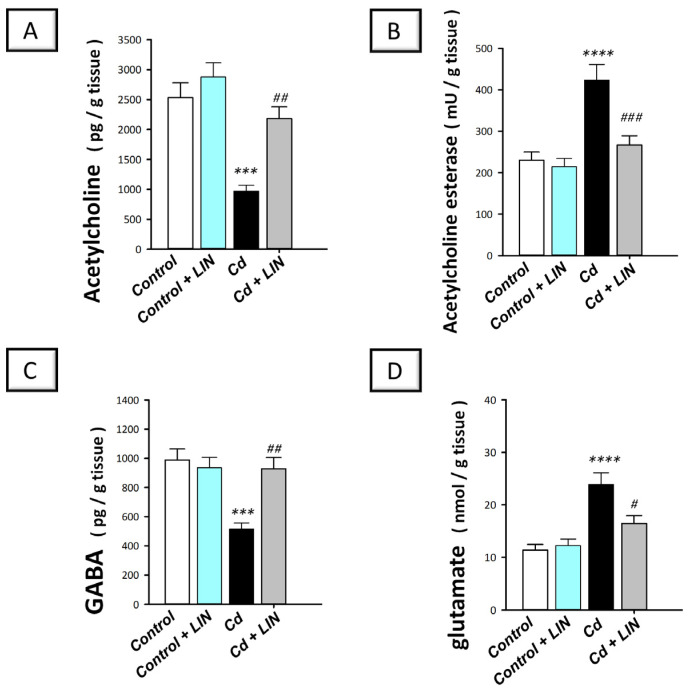
Linagliptin enhances acetylcholine and GABA and lowers acetylcholine esterase and glutamate in the hippocampi of cadmium-intoxicated rats. The level of acetylcholine neurotransmitter was increased (**A**) and acetylcholine esterase activity was lowered (**B**) by linagliptin in the hippocampi of cadmium-intoxicated rats. Moreover, the level of GABA was increased (**C**) while glutamate protein expression was lowered (**D**) by linagliptin. *N* = 6 in each group (mean ± standard error of the mean). A *p*-value of less than 0.05 was significant. *** p < 0.001, or **** p < 0.0001, compared to control; *^#^ p* < 0.05, *^##^ p* < 0.01, or *^###^ p* < 0.001, compared to cadmium (Tukey’s test for multi-comparisons and one-way ANOVA). Cd, cadmium chloride; LIN, linagliptin.

The cognitive decline has been tightly linked to the perturbation in the acetylcholine neurotransmitter in the hippocampi of rodents [29]. Hence, the levels of acetylcholine and acetylcholinesterase were examined in cadmium-intoxicated rats. Moreover, the hippocampal levels of GABA and neurotoxic glutamate were determined. Herein, significant differences existed among the experimental groups in the levels of acetylcholine (F (3, 20) = 16.72, *p* < 0.0001), acetylcholinesterase (F (3, 20) = 13.92, *p* < 0.0001), GABA (F (3, 20) = 10.70, *p* = 0.0002), and glutamate (F (3, 20) = 13.16, *p* < 0.0001), as illustrated in Figure 4A–D. Cadmium chloride led to a significant decline in hippocampal acetylcholine (*p* < 0.001), which reached 38.3% of the control values (Figure 4A). Meanwhile, cadmium triggered a significant increase in acetylcholine esterase activity (*p* < 0.0001) by 84.1% (Figure 4B). Additionally, cadmium chloride significantly diminished GABA (*p* < 0.001) by 48% and significantly elevated the neurotoxic glutamate (*p* < 0.0001) by 110% (Figure 4C,D). Compared to the cadmium-intoxicated group, these pathological changes were counteracted in response to linagliptin, as demonstrated by a significant increase in acetylcholine (*p* < 0.01) and GABA (*p* < 0.01) by 125.1% and 80.5%, respectively, alongside a significant reduction in acetylcholine esterase (*p* < 0.001) and glutamate (*p* < 0.05) by 36.9% and 31.1%, respectively. These findings reveal that the augmentation of acetylcholine and GABA, and curtailing acetylcholine esterase and glutamate are, at least partly, implicated in the improvement in the cognitive deficits triggered by cadmium in animals.

### 2.5. Linagliptin Attenuates the Pro-Oxidant Insult and Stimulates Hippocampal SIRT1/Nrf2/HO-1 Axis in Cadmium-Intoxicated Rats

**Figure 5:**
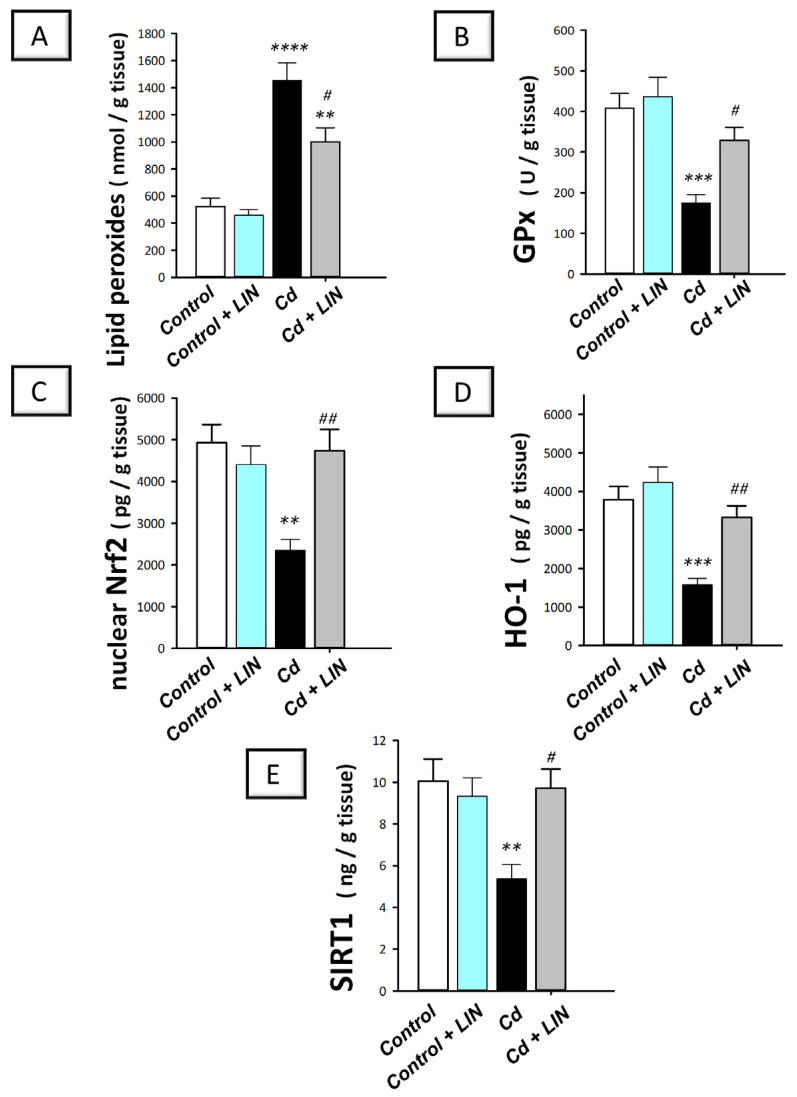
Linagliptin suppresses the hippocampal pro-oxidant response and stimulates SIRT1/Nrf2/HO-1 pathway in cadmium-intoxicated rats. Linagliptin lowers the lipid peroxides (**A**) and replenishes GPx (**B**), nuclear Nrf2 (**C**), HO-1 (**D**), and SIRT1 (**E**) in the hippocampi of cadmium-intoxicated rats. *N* = 6 in each group (mean ± standard error of the mean). A *p*-value of less than 0.05 was significant. ** p < 0.01, *** p < 0.001, or **** p < 0.0001, compared to control; *^#^ p* < 0.05, *^##^ p* < 0.01, compared to cadmium (Tukey’s test for multi-comparisons and one-way ANOVA). Cd, cadmium chloride; GPx, glutathione peroxidase; HO-1, heme oxygenase-1; LIN, linagliptin; Nrf2, nuclear factor erythroid-2-related factor-2.

Excessive hippocampal oxidative stress triggers neuronal degeneration and disruption of memory acquisition, culminating in cognitive impairment in rodents [3,4]. We examined hippocampal lipid peroxides and GPx activity. In addition, the antioxidant SIRT1/Nrf2/HO-1 pathway was explored in the hippocampi of rats. Herein, significant differences existed among the experimental groups in the levels of lipid peroxides (F (3, 20) = 26.08, *p* < 0.0001), GPx (F (3, 20) = 10.61, *p* = 0.0002), Nrf2 (F (3, 20) = 7.738, *p* = 0.0013), HO-1 (F (3, 20) = 13.74, *p* < 0.0001), and SIRT1 (F (3, 20) = 5.896, *p* = 0.0047), as illustrated in Figure 5A–E. Cadmium chloride led to a significant increase in hippocampal lipid peroxides (*p* < 0.0001) by 178.3% alongside a significant lowering in GPx activity (*p* < 0.001) by 57.2% (Figure 5A,B), revealing marked oxidative stress. This was associated with a significant reduction in nuclear Nrf2 (*p* < 0.01) by 52.3%, HO-1 (*p* < 0.001) by 58.3%, and SIRT1 (*p* < 0.01) by 46.5% (Figure 5C–E), demonstrating SIRT1/Nrf2/HO-1 pathway inhibition. Compared to the cadmium-intoxicated group, the hippocampal oxidative insult was mitigated in response to linagliptin. As evidence for these findings, lipid peroxides (*p* < 0.05) were significantly lowered by 31.1%, while GPx (*p* < 0.05), Nrf2 (*p* < 0.01), HO-1 (*p* < 0.01), and SIRT1 (*p* < 0.05) were significantly increased by 88.5%, 101.5%, 110.7%, and 80.9%, respectively. Together, these data reveal that linagliptin combated hippocampal oxidative stress and stimulated SIRT1/Nrf2/HO-1 axis, favorable events that are, at least partly, implicated in the improvement in cognitive deficits triggered by cadmium in animals.

### 2.6. Linagliptin Counteracts Neuronal Apoptosis in the Hippocampi of Cadmium-Intoxicated Rats

**Figure 6:**
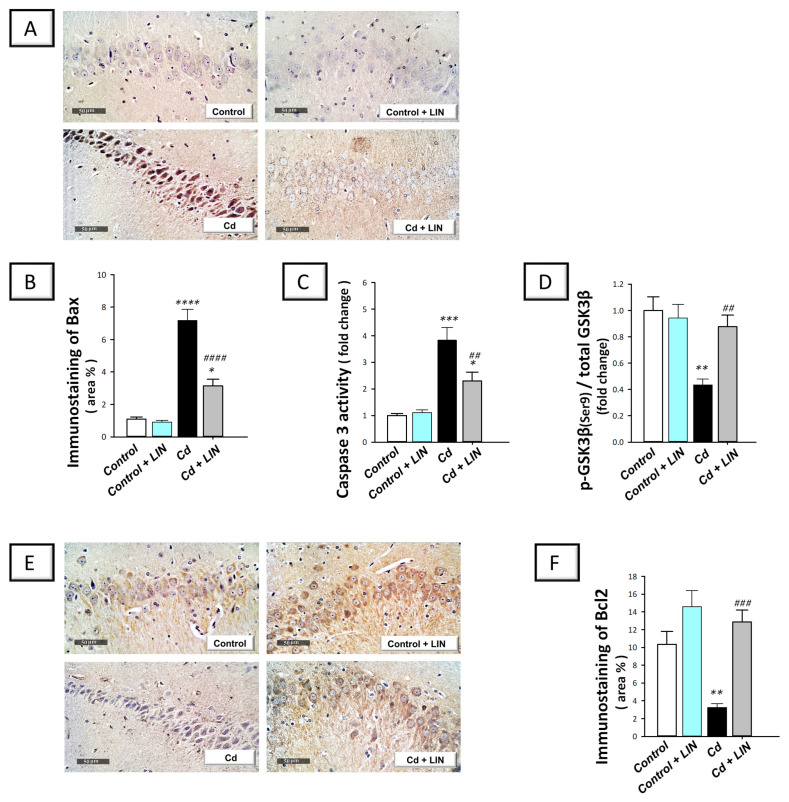
Linagliptin inhibits hippocampal pro-apoptotic events in cadmium-intoxicated rats. Linagliptin downregulated Bax protein expression in hippocampal CA3 region (**A**,**B**), lowered caspase 3 activity (**C**), and increased the protein expression of the inactive form of the pro-apoptotic kinase GSK-3β (Ser9) (**D**) in the hippocampi of cadmium-intoxicated rats. (**A**) Representative immunohistochemistry images of Bax in hippocampal CA3 region (scale bar: 50 µm). (**B**) Bax protein quantitation (area %; 6 non-overlapping microscopic fields). Moreover, linagliptin upregulates Bcl-2 in hippocampal CA3 region in cadmium-intoxicated rats. (**E**) Representative immunohistochemistry images in hippocampal CA3 region (scale bar: 50 µm). (**F**) Bcl-2 protein quantitation (area %; 6 non-overlapping microscopic fields). *N* = 6 in each group (mean ± standard error of the mean). A *p*-value of less than 0.05 was significant. * p < 0.05, ** p < 0.01, *** p < 0.001, or **** p < 0.0001, compared to control; *^##^ p* < 0.01, *^###^ p* < 0.001, or *^####^ p* < 0.0001, compared to cadmium (Tukey’s test for multi-comparisons and one-way ANOVA). Bax, Bcl-2-associated x protein; Bcl-2, B-cell lymphoma-2 protein; Cd, cadmium chloride; GSK-3β, glycogen synthase kinase—3 beta; LIN, linagliptin.

Growing evidence has revealed that cadmium activates the apoptotic machinery culminating in neuronal cell death in vitro [11]. In rodents, apoptosis has been linked to neurotoxicity and neuronal degeneration in cadmium-induced neuronal damage [4,5]. Hence, we examined whether combating apoptosis is one of the underlying mechanisms for the promising effects of linagliptin. Herein, we detected Bax and Bcl-2 protein expression in the hippocampi of rats using immunohistochemistry. Meanwhile, caspase-3 activity and the protein expression of phosphorylated GSK-3β(Ser9)/total GSK-3β were determined. Herein, significant differences existed among the experimental groups in the levels of Bax (F (3, 20) = 50.10, *p* < 0.0001), caspase 3 (F (3, 20) = 19.46, *p* = 0.0002), and p-GSK-3β(Ser9) (F (3, 20) = 8.588, *p* = 0.0007) alongside Bcl2 (F (3, 20) = 13.38, *p* < 0.0001), as illustrated in Figure 6B–D,F, respectively. The pro-apoptotic Bax protein expression was elevated by 553.2% in response to cadmium chloride (Figure 6A,B) with a significant reduction (*p* < 0.01) in Bcl-2 protein expression by 68.5% (Figure 6F), revealing augmented neuronal pro-apoptotic events. In tandem, hippocampal caspase 3 activity was significantly elevated (*p* < 0.001) by 283% (Figure 6C) and the inactive form of the pro-apoptotic kinase GSK-3β (Ser9) was significantly lowered (*p* < 0.01) by 56.5% (Figure 6D). Compared to the cadmium-intoxicated group, the pro-apoptotic machinery was dampened in response to linagliptin, as demonstrated by a significant decline in Bax protein levels (*p* < 0.0001) and caspase 3 activity (*p* < 0.01) by 56.1% and 39.9%, respectively, alongside a significant increase in Bcl-2 (*p* < 0.001) and GSK-3β (Ser9) (*p* < 0.01) by 293.8% and 101.7%, respectively. Of note, the detected lowering in Bcl2 immunostaining area % could be due to neuronal loss; shrinkage of the neuronal soma, which has been reported as a common pathological outcome in Alzheimer’s disease; or both [30]. In this context, the immunostaining area % does not differentiate between neuronal loss and soma shrinkage. Overall, these findings reveal that linagliptin combated hippocampal neuronal apoptotic cell death. These favorable anti-apoptotic events are, at least partly, implicated in the improvement in the cognitive deficits triggered by cadmium in animals.

### 2.7. Linagliptin Enhances the Pro-Autophagy Events in the Hippocampi of Cadmium-Intoxicated Rats

**Figure 7:**
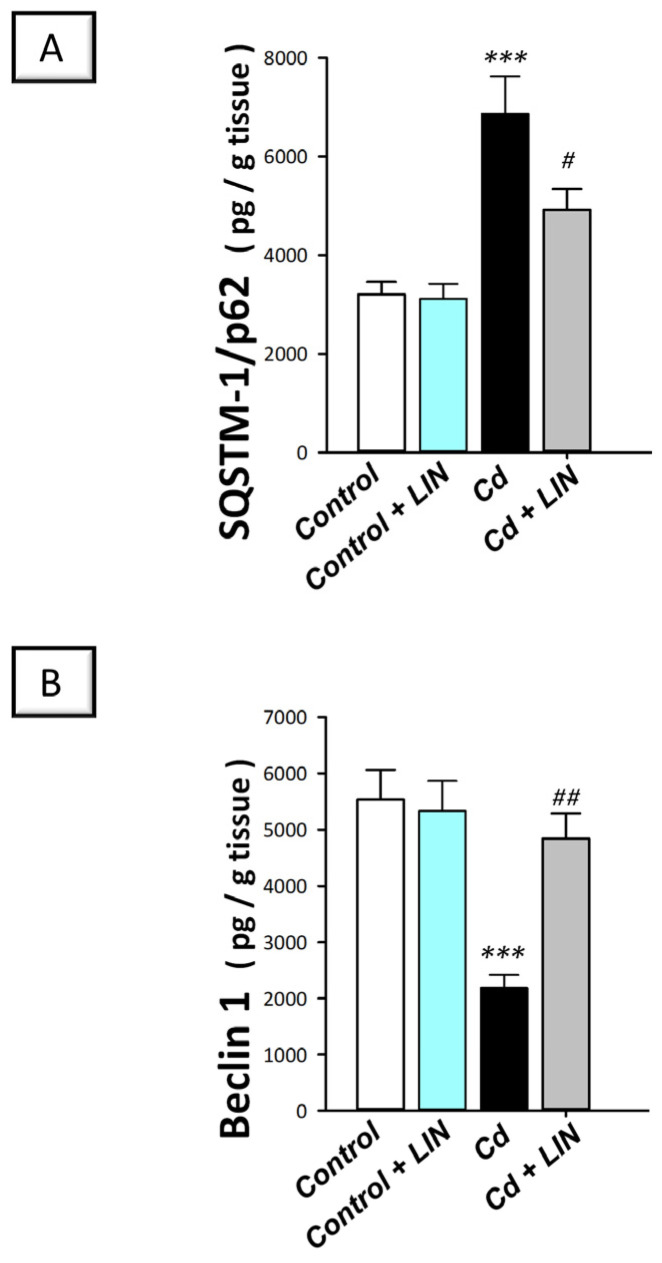
Linagliptin activates autophagy in the hippocampi of cadmium-intoxicated rats. This was demonstrated by SQSTM-1/p62-diminished accumulation (**A**) and Beclin-1-upregulated protein expression (**B**) in the hippocampi of cadmium-intoxicated rats. *N* = 6 in each group (mean ± standard error of the mean). A *p*-value of less than 0.05 was significant. *** p < 0.001, compared to control; *^#^ p* < 0.05, *^##^ p* < 0.01, compared to cadmium (Tukey’s test for multi-comparisons and one-way ANOVA). Cd, cadmium chloride; LIN, linagliptin; SQSTM-1/p62, sequestome sequestosome-1/protein 62.

In the context of autophagy flux, contradictory results have been reported in the literature regarding the effects of cadmium. Defective autophagy flux has been previously characterized in Neuro-2a cells [8], rat primary cortical neurons [10], PC12, and primary murine neurons [11]. In contrast, the stimulation of autophagy has been demonstrated in vitro in hippocampal neuronal TH22 cells [9]. More importantly, the in vivo effect of cadmium on the hippocampi of rats has not been sufficiently investigated. Thus, we examined autophagy in the hippocampi of cadmium-intoxicated rats by determining the protein expression of SQSTM-1/p62, a well-characterized marker of impaired autophagy. In fact, accumulation of the later protein indicates defective autophagosome degradation and autophagy inhibition [12]. In addition, the hippocampal levels of Beclin 1 were explored as a marker of autophagy flux enhancement [31]. Herein, significant differences existed among the experimental groups in the levels of SQSTM-1/p62 (F (3, 20) = 13.92, *p* < 0.0001) and Beclin 1 (F (3, 20) = 11.80, *p* = 0.0001), as illustrated in Figure 7A,B. Cadmium chloride significantly (*p* < 0.001) elevated SQSTM-1/p62 protein levels by 114.5% (Figure 7A) and significantly (*p* < 0.05) diminished Beclin 1 (*p* < 0.001) by 60.5% (Figure 7B), revealing impaired hippocampal autophagy. Compared to the cadmium-intoxicated group, the pro-autophagy events were enhanced in response to linagliptin. In perspective, SQSTM-1/p62 protein levels were significantly diminished (*p* < 0.05) by 28.3% and Beclin 1 was significantly elevated (*p* < 0.01) by 121.4%. These findings show that linagliptin’s autophagy augmentation is, at least partly, implicated in the improvement in the cognitive deficit triggered by cadmium in animals.

### 2.8. Linagliptin Stimulates Hippocampal AMPK/mTOR Pathway in Cadmium-Intoxicated Rats

**Figure 8:**
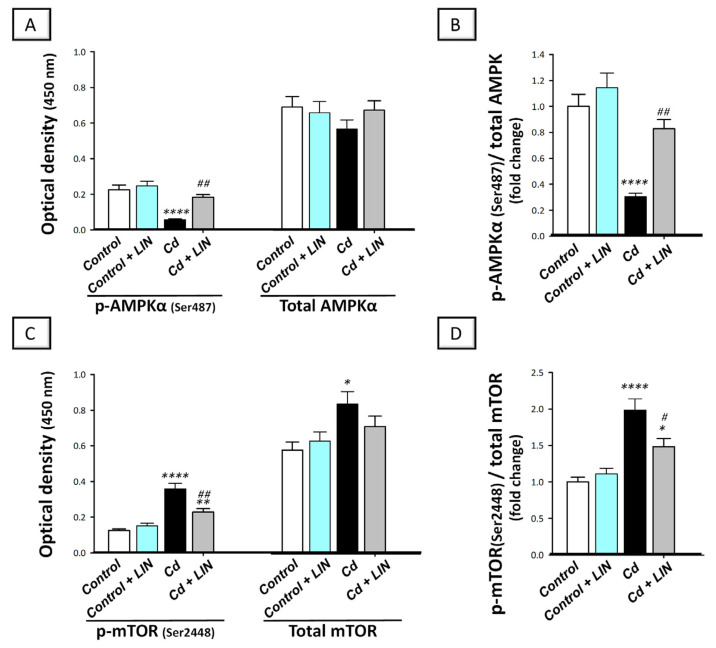
Linagliptin stimulates hippocampal AMPK/mTOR pathway in cadmium-intoxicated rats. Herein, stimulation of AMPK/mTOR pathway by linagliptin in cadmium-intoxicated animals was reflected by the elevation in p-AMPK/AMPK ratio (**B**), and reduction in p-mTOR/mTOR ratio (**D**). Of note, the individual O.D. of p-AMPK(Ser487) and total AMPK are shown in (**A**), whereas the individual O.D. of p-mTOR(Ser2448) and total mTOR are shown in (**C**). For each experimental group, the mean optical density of either p-AMPK or p-mTOR was divided by the corresponding mean optical density of the total AMPK or total mTOR, respectively. This was followed by setting the mean control value to 1. *N* = 6 in each group (mean ± standard error of the mean). A *p*-value of less than 0.05 was significant. * p < 0.05, ** p < 0.01, or **** p < 0.0001, compared to control; ^#^ *p* < 0.05, or ^##^ *p* < 0.01, compared to cadmium (Tukey’s test for multi-comparisons and one-way ANOVA). AMPK, 5′adenosine-monophosphate-activated protein kinase/ mammalian target of rapamycin; Cd, cadmium chloride; LIN, linagliptin; mTOR, mammalian target of rapamycin.

Autophagy progression is positively impacted by AMPK/mTOR pathway, according to evolving evidence [12]. Herein, we explored the in vivo effect of cadmium on the autophagy-linked AMPK/mTOR pathway in the hippocampi of cadmium-intoxicated rats. The phosphorylation step is the main event for controlling the activity of AMPK and mTOR [3,9,12]; thus, the data were expressed as the phosphorylated form of AMPK or mTOR to the total form of AMPK or mTOR, respectively. Significant differences existed among the experimental groups in the ratio of p-AMPK/total AMPK (F (3, 20) = 19.53, *p* < 0.0001) and p-mTOR/total mTOR (F (3, 20) = 17.04, *p* < 0.0001), as illustrated in Figure 8. Cadmium chloride significantly diminished p-AMPK/total AMPK ratio (*p* < 0.001)—a well-recognized activator of the autophagy machinery—by 69.5% (Figure 8B) and significantly elevated the p-mTOR/total mTOR ratio (*p* < 0.001)—a well-characterized autophagy inhibitory signal—by 98.5% (Figure 8D), demonstrating AMPK/mTOR pathway inhibition. Compared to the cadmium-intoxicated group, AMPK/mTOR pathway stimulation was detected in response to linagliptin. In perspective, the p-AMPK/total AMPK ratio (*p* < 0.01) was significantly boosted by 171.6%, and the p-mTOR/total mTOR ratio was significantly diminished (*p* < 0.05) by 25.3%. Together, these favorable events are, at least partly, implicated in cleansing neuronal misfolded proteins and in the improvement in the cognitive deficit triggered by cadmium in animals.

## 3. Discussion

**Figure 9:**
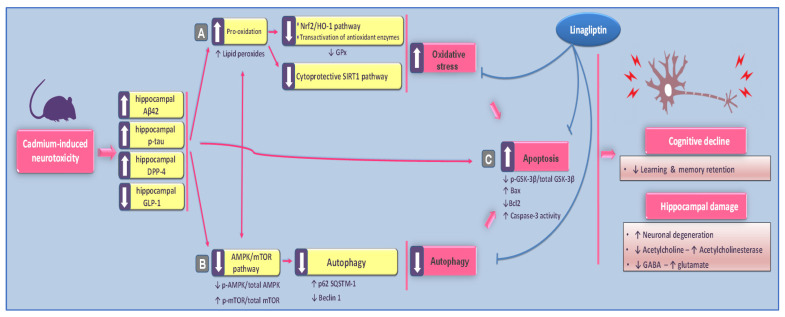
A summary of the proposed molecular pathways for linagliptin’s neuroprotection against the cognitive impairment induced by cadmium in rats. Based on the current findings, linagliptin ameliorated cadmium-induced retention/recognition memory disruption and neuronal degeneration by augmenting the hippocampal GLP-1 levels. The dampened cadmium-induced neurotoxicity was manifested by lowered hippocampal Aβ42 and p-tau noxious signals alongside augmenting acetylcholine/GABA and lowering neurotoxic glutamate. At the molecular level, these favorable effects were brought by (**A**) combating hippocampal oxidative insult and stimulation of SIRT1/Nrf2/HO-1 cytoprotective pathway; (**B**) stimulation of the pro-autophagy response and activation of AMPK/mTOR cascade; (**C**) reversal of hippocampal apoptotic machinery and inactivation of the pro-apoptotic kinase GSK-3β. Arrows: an arrow with a solid line denotes activation; an arrow with a blunt line indicates inhibition.

The present study revealed the in vivo evidence for linagliptin’s neuroprotective effects against cadmium-evoked cognitive impairment in rats. At the molecular and cellular levels, linagliptin combated cadmium-evoked neurotoxicity as manifested by dampening Aβ42 and p-tau noxious signals and increasing acetylcholine/glutamate ratio in the hippocampi of rats. In perspective, multi-pronged mechanisms were targeted by linagliptin, which suppressed the oxidative injury and activated SIRT1/NRF2/HO-1 pathway. Additionally, linagliptin activated hippocampal-AMPK/mTOR-driven autophagy and inhibited the pro-apoptotic events in animals (Figure 9).

Cadmium is a widely existing environmental pollutant that triggers diverse noxious health effects including cognitive impairment and Alzheimer-like manifestations. Postmortem studies in humans revealed higher cadmium content in Alzheimer’s brain tissue compared to age-matched healthy control specimens [1]. Several epidemiological studies revealed that repeated exposure to cadmium is tightly linked to diminished cognitive function in the human population [2]. Ample evidence has demonstrated that cadmium readily crosses the blood–brain barrier, thereby accumulating in several brain regions, particularly the hippocampus, which regulates memory functions [3,4]. Hence, cadmium has been reported to disrupt spatial learning and memory in rodents [3]. The present study is in harmony with these data, showing that cadmium impaired the retention and recognition memory in rats. At the cellular and molecular level, accumulation of Aβ42 in the hippocampi has been regarded as a hallmark in Alzheimer’s pathogenesis due to its high tendency for aggregation and consequent neurotoxicity [3,4,5]. Cholinergic neuron toxicity is another suggested mechanism of cadmium-evoked cognitive deficits. Cadmium exposure has been linked to cholinergic cell death and a decline in hippocampal cholinergic neurotransmission [1,32]. In the same context, Aβ42 has been reported to increase glutamate levels, triggering activation of multiple intracellular signaling and culminating in marked neurotoxicity and neuronal cell death [33]. Equally important, the deposition of Aβ42 triggers tau phosphorylation and neurofibrillary tangle accumulation, culminating in neuronal cell death, synaptic damage, and memory disruption [4,12].

Ample evidence revealed that modalities that dampen the neurotoxic Aβ42 and p-tau and enhance the cholinergic transmission in the hippocampi of rodents have been proven to halt the progression of cognitive decline and associated spatial learning/memory deficits [29,34]. The present study is consistent with these data by showing that linagliptin improved the cognitive impairment caused by cadmium by downregulating hippocampal Aβ42 and p-tau, augmenting acetylcholine, and lowering the noxious glutamate. In the same context, rodent models of cognitive impairment have demonstrated that linagliptin improves cognitive performance in streptozotocin-induced diabetic dementia [13], cerebral ischemia in type 2 diabetes [18], and high-fat-evoked cognitive deficits [19]. Moreover, the dampening of acetylcholine esterase has been proven as an effective approach against Aβ-induced neuronal injury [29].

The current study demonstrated an enhanced hippocampal oxidative injury and inhibition of SIRT1/Nrf2/HO-1 cascade. These noxious events were counteracted by linagliptin. Virtually, linagliptin’s marked antioxidant actions have been previously elucidated in rodent models of cerebral ischemia in type 2 diabetes [18] and high-fat-evoked cognitive deficit [19]. In addition, stimulation of Nrf2/HO-1 antioxidant cascade mediated linagliptin’s antioxidant actions for combating the pathological manifestations of diabetic kidney disease [25] and inflammatory bowel disease [15]. Importantly, the observed linagliptin-induced amelioration of learning and memory impairment was associated with SIRT1/Nrf2/HO-1 axis stimulation. In perspective, the cytoprotective signal SIRT1 has demonstrated favorable actions for combating Alzheimer’s manifestations [6]. In APP/PS1 transgenic mice, SIRT1 upregulation has been associated with lowered plaque deposition, p-tau accumulation, and improved behavioral phenotype [6,35]. Interestingly, the interplay between SIRT1 and the autophagy-linked AMPK/mTOR signaling has been characterized where SIRT1 negatively regulates mTORC1 via interacting with TSC1/2, culminating in marked neuroprotection in senile mice [36]. In addition, SIRT1 has been proven to increase the nuclear translocation/transcriptional activity of Nrf2, an outcome that favors its binding to the antioxidant-response elements (AREs) with consequent stimulation of the antioxidant HO-1 [5]. Interestingly, augmentation of Nrf2 signaling has been reported to mediate the amelioration of cadmium-induced neurotoxicity in PC12 in vitro and brain oxidative damage/neuronal insult in the mouse cortex [37].

When neurons are exposed to high levels of cadmium, mitochondrial apoptosis machinery dominates as the programmed death pathway [4,5]. Moreover, rats exposed to cadmium-induced neurotoxicity were shown to exhibit an increased Bax/Bcl-2 ratio, indicating activation of the intrinsic apoptotic pathway [4,5,7]. Evidence demonstrated that cadmium disrupts the mitochondrial membrane potential, stimulating apoptosis as seen by an intensified activity of the executioner caspase-3 [7]. In accordance with previous data, the current study depicted that cadmium-induced cognitive impairment in rats was associated with exaggerated neuronal apoptosis as seen by upregulated expression of hippocampal Bax together with downregulation of the anti-apoptotic Bcl-2. GSK-3β is a pro-apoptotic kinase that is switched on in response to Aβ neuronal accumulation [38]. The Aβ-triggered GSK-3β hyperactivity has been reported to instigate neuronal apoptosis through the mitochondrial pathway, culminating in neuronal loss and memory dysfunction [4,38,39]. Herein, significant suppression of hippocampal apoptotic events and enhancement of neuronal survival were elicited by linagliptin. These events favor recovery from neuronal injury and boost memory acquisition/learning ability [4]. Notably, the literature has characterized evident anti-apoptotic actions of linagliptin that mediated its beneficial actions against diabetic renal injury as proven by upregulating Bcl-2 and diminishing caspase-3 [24]. In cultured podocytes, linagliptin abrogated high-glucose-induced apoptosis via IRS1/AKT pathway modulation [25]. Given the fact that oxidative stress signals are major activators of neuronal apoptotic machinery [4,7], the current study findings and the well-reported [13,14,18,23] anti-oxidant features of linagliptin may advocate its promising anti-apoptotic actions. Additionally, linagliptin’s observed activation of hippocampal autophagy events can attenuate the pro-apoptotic machinery by neuronal clearance of damaged mitochondria/ROS, favoring pro-survival signals [4,11].

In neurons, basal autophagy levels are crucial for cellular survival and homeostasis [8]. Ample evidence characterized impaired autophagy in neurodegenerative disorders and associated neuronal cell loss. In perspective, in vitro studies on cadmium-induced neurotoxicity demonstrated defective autophagy in Neuro-2a cells [8], rat primary cortical neurons [10], rat pheochromocytoma (PC12), and primary murine neurons [11]. In fact, disrupted clearance of the autophagic vacuoles that serve as intracellular reservoirs for Aβ production has been associated with excessive deposition of the neurotoxic amyloid aggregates in Alzheimer’s brains [40]. Conceptually, autophagy impairment proven by the accumulation of neuronal autophagosomes has been reported to initiate neuronal apoptosis [41]. In this regard, when cadmium-evoked neuronal cell stress exceeds a critical intensity threshold or duration, the apoptotic machinery is activated, confirming the sequential activation of autophagy and apoptosis [11,41]. In harmony with these reports, the current findings showed that cadmium-triggered cognitive impairment was associated with hippocampal Beclin 1 decline and SQSTM-1/p62 accumulation, confirming impaired autophagy. Virtually, the autophagy flux describes the sequence of events that rid the neurons of damaged proteins and organelles, such as mitochondria. These include autophagosome synthesis, autophagic cargo delivery to lysosomes, and ultimately destruction/recycling of the damaged macromolecules/organelles [31]. In the context of autophagy flux, disrupted autophagy is indicated by SQSTM-1/p621 accumulation since the active process of autophagy degrades this protein. In tandem, Beclin1 has been regarded as a pro-autophagy marker owing to its involvement in autophagosome synthesis [12,31]. It is noteworthy that the literature characterizes some in vivo models that show autophagy stimulation in cadmium-induced hippocampal dysfunction [9], which contradicts the present data. Several factors can contribute to this controversy, including the difference in animal species (rat vs. mouse), cadmium exposure duration, as well as the severity of hippocampal damage. In perspective, mild neuronal injury can stimulate autophagy to clear the neurotoxic signals while severe hippocampal damage instigates overactive autophagy and neuronal death under excitotoxic-stimuli-linked neurodegeneration [5,9].

Autophagy stimulation plays a fundamental role in counteracting cognitive deficits via the removal of tau and aggregated neurofibrillary tangles [9]. Interestingly, agents that stimulate autophagy flux activation such as rapamycin have been proven to rescue tau pathology/cognitive impairment in experimental Alzheimer’s disease [9,12]. Herein, increasing Beclin1 levels in response to linagliptin were consistent with these data, as well as a lower accumulation of the negative autophagy marker SQSTM-1/p621. Notably, a previous report in a rat model of diabetic nephropathy demonstrated linagliptin’s ability to stimulate autophagy in vivo [24]. It was also shown that linagliptin upregulated Beclin1 and LAMP-1 expression in the glomeruli of db/db mice, confirming autophagy stimulation [24]. Interestingly, linagliptin’s antioxidant features [13,14,18,23] may also drive its pro-autophagic actions in cadmium-induced neurotoxicity since excessive pro-oxidant events are linked to autophagy impairment [5].

In neurodegenerative diseases, autophagy has been reported to be stimulated in response to AMPK/mTOR pathway activation [9]. This is associated with Aβ and p-tau aggregate clearance with abatement of cognitive impairment in rodents [12]. Lining up with the previous findings, linagliptin lowered p-mTOR (Ser2448)/total mTOR and elevated p-AMPK(Ser487)/total AMPK ratio, confirming AMPK/mTOR axis stimulation. By acting as a low-energy sensor, AMPK plays an essential role in homeostasis against stress conditions in brain tissue by inhibiting mTORC1 via TSC2 and acting as a negative regulator of mTOR [3,12]. Interestingly, activators of AMPK, including resveratrol and quercetin, have been proven to clear Aβ deposition, thereby dampening neurotoxicity in rodents [12]. Meanwhile, the inhibition of mTOR, a negative regulator of autophagy, has been associated with marked neuroprotection and amelioration of cognitive dysfunction [12,31].

## 4. Materials and Methods

### 4.1. Chemicals

Boehringer Ingelheim (Trajenta^®^; Berlin, Germany) provided linagliptin as a generous gift, while cadmium chloride was purchased from Sigma-Aldrich (Cat. # 202908; St. Louis, MO, USA). Each assay includes a description of the kit/reagent supplier. All remaining chemicals were acquired in the highest purity grade.

### 4.2. Rats, Housing, and Ethical Statement

The animals used for the current study were adult male Wistar albino rats (weight: 180–200 g; age: 10 weeks; Breeding Unit of the Egyptian Drug Authority (EDA), Giza, Egypt). Rats were maintained under controlled experimental conditions (housing in polycarbonate cages, 12L/12D lighting cycle, 50% humidity, and 21–24 °C temperature). Ten days before the start of the experimental protocol, animals were allowed an acclimatization span with ad libitum access to laboratory chow and drinking water.

The Experimental and Clinical Studies Research Ethics Committee at EDA approved the protocol for the study (Approval ID: NODCAR/I/8/2022). Herein, we followed the Laboratory Animal Care and Use Guide (US-NIH, Publication # 85-23) for animal handling and experimental methodology.

### 4.3. Establishing Preclinical Animal Model and Experimental Design

Since sample size in animal behavioral studies is a crucial factor that affects the reliability and statistical power of the obtained results, we used power analysis for sample size calculation [42]. In power analysis, the effect size was determined based on the previously published data [3], with power = 0.8 and α = 0.05, and the total number of animals was determined as 40, which were randomly divided into 4 experimental groups (each contains 10 rats). The animals were carefully handled to minimize stress and suffering. Table 1 depicts the experimental design. Notably, random animal distribution was applied by a blinded technician.

### 4.4. Evaluation of the Cognitive Function (Learning and Memory Acquisition)

The behavioral tests were conducted by blinded researchers unaware of the study design or experimental group labels to prevent bias.

#### 4.4.1. Morris Water Maze (MWM)

The MWM paradigm investigates the spatial learning and memory retention of animals [27]. A circular water tank (150 cm in diameter and 60 cm high) containing water (ambient temperature) to a depth of 40 cm served as the testing equipment. In the midpoint of one quadrant, a transparent escape platform (8 cm diameter; Plexiglas) was kept invisible underwater by placing it 2 cm below the water’s surface. At the end of the experimental period, the animals were permitted 3 days of training (4 training trials each day; 1 min each) using a concealed platform in one quadrant and three rotating beginning quadrants [45]. During these training (acquisition) trials, rats were kept free to find the hidden platform for 1 min. Once the animal found the platform, it was permitted to settle for 30 s on it. The animal was placed on the platform for 60 s if it failed to find the hidden platform within 1 min. During the probe test (retrieval trial) on the 4th day, the animals were allowed to explore the pool for 1 min. The time spent in the target quadrant was an indicator of memory consolidation.

#### 4.4.2. Y-Maze Test

The Y maze included 3 identical arms (labeled as A, B, and C) located at equal angles as described in [46]. For each arm, it measured 47 cm in length and 20 cm in height. For the trial session, each animal was positioned in the center of the device and allowed to freely roam through the maze with one arm closed for 10 min. Hence, the animal was allowed to explore 2 arms only. After 1 h, the test session was applied where the closed arm was opened, and each animal was allowed to freely explore the 3 arms. In this regard, the animal with good spatial memory enters the novel (unexplored) arm more frequently than the other arms. For each animal, a recording of the time spent in each arm was performed and a calculation of the ratio of time spent in the new arm/old arm was applied. The arm entry was described as the entry of all four paws of animals into one arm. Cleaning of the apparatus with 70% ethanol was performed after each trial/test to remove potential bias due to odor cues.

#### 4.4.3. Novel Object Recognition Test (NORT)

Through the novel object recognition test, we examined the recognition memory of animals that assesses their spontaneous tendency to explore novel objects [28]. Herein, the novel object arena was composed of an open-field 80 × 80 × 40 cm box with white background and black grid lines. In the experimental room, the box was illuminated, and no shadows were reflected on it. Habituation, familiarization, and testing stages were applied during the test. First, habituation was applied by allowing animals to freely explore the empty box for 10 min. After 24 h, the training (familiarization) stage was conducted for 5 min during which animals were allowed to explore 2 identical non-toxic objects located in a fixed position (A1 and A2). Exploration was defined as touching or approaching the object with the animal nose for a distance lower than or equal to 2 cm. Elimination took place for animals that did not explore the objects during the training trial. Cleaning of the box and objects with 70% ethanol was applied after each animal to remove potential bias due to the odor cues. Twenty-four hours after the training session, the testing stage was executed where a novel object (B) with a similar material/color but different shape was added instead of one of the two familiar objects, and animals were allowed to freely explore for 5 min. We calculated the discrimination ratio using the formula = time spent examining novel objects/total time spent exploring both familiar and novel objects [28].

### 4.5. Collecting the Brain Tissue

Twenty-four hours after the completion of cognitive tests, blood was withdrawn for serum separation, and rats were sacrificed by decapitation. Instantly, brains were collected, and the hippocampi were dissected and instantly stored at −80 °C for biochemical determinations. An ice-cold lysis buffer (200 mM NaCl, 5 mM EDTA, 10 mM Tris, 10% glycerol (pH 7.4)) containing phosphatase/protease inhibitors (1 μg/mL leupeptin; 28 μg/mL aprotinin, and 1 mM PMSF) was used to homogenize part of the hippocampus for ELISA measurements. After homogenate centrifugation (10,000× *g*; 15 min), the supernatant was kept at −80 °C for further processing. For the remaining determinations, phosphate-buffered saline (pH 7.4) was used to homogenize another part of the hippocampus. Histopathology/immunohistochemistry was performed on 4 randomly selected brains from each group.

### 4.6. Serum Glucose and Hippocampal DPP-4 and GLP-1

A HUMAN colorimetric kit (Cat. # BD184; HUMAN Diagnostics, Wiesbaden, Germany) was used to determine serum glucose levels under the guidance of the provider. A final color reading was taken at a wavelength of 500 nm. The contents of dipeptidyl peptidase-4 (DDP-4; Cat. # E0226Ra; Bioassay Technology Laboratory, Shanghai, China) and glucagon-like peptide-1 (GLP-1; Cat. # SL0304Ra; SunLong Biotech. Company, Ltd., Hangzhou, China) were quantified by commercially available ELISA kits as described by the vendor. A final color reading was taken at a wavelength of 450 nm.

### 4.7. Histopathology

To avoid bias, the histopathology processing/examination was carried out by a blinded histologist. Formalin-fixed brain sections were processed for paraffin-embedding by washing, dehydration by alcohol, and clearing in xylene as previously described [34]. Paraplast tissue embedding media were used to embed the sections. Five-micrometer sagittal brain sections were stained by the classic hematoxylin and eosin (H-E) protocol and a light microscopy examination of the morphological aberrations was conducted (Leica Microsystems, Wetzlar, Germany). In H-E, 4 specimens were examined per experimental group and 6 non-overlapping fields were photographed [47,48].

The neuropathological damage in terms of pyknosis and microglial cell infiltration was quantified with the aid of a 0–4 scoring system as described earlier [49,50]. In brief, the absence of specific lesions was scored as zero, the presence of the neuropathological lesion in less than 10% of the area was scored as 1, the presence of the neuropathological lesion in 10–40% of the area was scored as 2, the presence of the neuropathological lesion in 40–60% of the area was scored as 3, and the presence of the neuropathological lesion in more than 60% of the area was scored as 4.

### 4.8. Immunohistochemistry

Bias was avoided in immunohistochemistry by keeping the specimen identity anonymous. Bax and Bcl-2 protein expressions were examined by immunohistochemical staining of the target protein in paraffin-embedded brain sections (5 µm) as reported in [51]. In brief, deparaffinized sections were processed for antigen retrieval. In a humidified chamber, section blockade with 5% BSA was applied; then, overnight application of the primary antibodies was performed in the fridge: anti-Bcl-2 (Cat. # PA1-30411; 1:100 dilution) or anti-Bax (Thermo Fisher Scientific, Waltham, MA, USA; Cat. # 33-6600; 1:100 dilution). Section washing with PBS was conducted and HRP-labeled secondary antibody incubation was performed for 20 min (EnVision kit, Dako, Copenhagen, Denmark). A similar procedure was followed for negative control sections, except that the primary antibody incubation was omitted. Visualization of the brown immunostaining was performed using 3,3′-diaminobenzidine (DAB) chromogen for 15 min and section counterstaining with hematoxylin was conducted. The immunostaining was photographed under a light microscope (Leica Microsystems, Wetzlar, Germany). To compensate for irregularities in the illumination of the microscopic field, shading error correction was performed before measurements. We imaged six non-overlapping fields in each experimental group. Fiji ImageJ^®^ (NIH, Bethesda, MD, USA; version 1.51r) was used to calculate the brown staining area % on positive areas of the digital images. In order to determine the average area % of immunostaining, the RGB input image was separated into three eight-bit images using the color deconvolution 2 plugin, and the color analysis of the brown immunostaining was conducted on the DAB image. Herein, a single focal plane was used to perform all the measurements, which were conducted on equalized images. For all samples, threshold settings were fixed, and the same settings were applied during all quantifications and image analysis to gather unbiased and valid results. In this regard, we adjusted the maximum threshold value so that the background signal was omitted without removing the true DAB signal. To obtain an average maximum threshold value, the maximum threshold was determined for at least five images [52]. Notably, the observed decline in the immunostaining area % is likely due to the loss of neurons, shrinkage of neuronal soma—a pathological outcome during neurodegeneration—or both. In fact, measuring the area % does not differentiate between neuronal loss and soma shrinkage [30].

### 4.9. Measurement of Hippocampal Aβ42, p-Tau, and Neurotransmitters

The hippocampal protein expression of the neurotoxic amyloid-β (Aβ42; Cat. # E-EL-R1402; Elabscience, Wuhan, China) and phosphorylated tau (p-tau; Cat. # ER1304; Fine Test, Wuhan Fine Biotech Co., Ltd., Wuhan, China) were quantified by commercially available ELISA kits as described by the provider. For both kits, the final color reading was taken at a wavelength of 450 nm. Regarding hippocampal neurotransmitters, the content of acetylcholine (ACh) was assayed using Cloud-Clone Corp. ELISA kit (Cat. # CEA912Ge; Cloud-Clone Corp., Houston, TX, USA), while the activity of acetylcholine esterase (AChE) was quantified using Kamiya acetylcholinesterase fluorescent activity kit (Cat. # KT-708; Kamiya Biomedical, Seattle, WA, USA). The fluorescent emission was read at 510 nm with excitation at 370 nm. Moreover, the hippocampal level of glutamate was quantified by AFG Bioscience ELISA kit (Cat. # EK721805; AFG Bioscience, Northbrook, IL, USA), whereas the hippocampal level of gamma amino butyric acid (GABA) was measured by BioVision ELISA kit (Cat. # E4457-100; BioVision Incorporated, Milpitas, CA, USA). For glutamate and GABA determination, the final color reading was taken at a wavelength of 450 nm.

### 4.10. Markers of Autophagy and Apoptosis

RayBiotech ELISA kit was used for the quantification of the phosphorylated and total forms of AMPK under the guidance of the vendor (Cat. # PEL-AMPKA-S487-T; Norcross, GA, USA). Likewise, ELISA kits from Cell Signaling were used to quantify phosphorylated and total forms of mTOR (Cat. # 7976C for p-mTOR[Ser2448] and Cat. # 7974C for pan mTOR, Cell Signaling Technology, Danvers, MA, USA). The final color reading was taken at a wavelength of 450 nm. As guided by the vendors, the expression of the results for the changes in p-AMPK[Ser487] and p-mTOR[Ser2448] was illustrated as the ratio of p-AMPK[Ser487])/total AMPK and p-mTOR[Ser2448]/total mTOR, respectively. For each experimental group, the mean optical density of either p-AMPK or p-mTOR was divided by the corresponding mean optical density of the total AMPK or total mTOR, respectively. This was followed by setting the mean control value to one [53]. SQSTM-1/p62 and Beclin1 protein levels were quantified using the SunLong Biotech. ELISA kit (Cat. # SL1363Ra; SunLong Biotech. Company, Ltd., Hangzhou, Zhejiang, China) and the AFG Bioscience ELISA kit (Cat. # EK720982; AFG Bioscience, Northbrook, IL, USA), respectively, under strict adherence to the provider protocol. For SQSTM-1/p62 and Beclin1, the final color reading was taken at a wavelength of 450 nm.

Under strict adherence to the instructions provided by the vendor, a colorimetric assay made by Sigma-Aldrich (Cat. # CASP-3-C; Sigma-Aldrich, St. Louis, MO, USA) was used to determine caspase-3 activity. The final color reading was taken at a wavelength of 405 nm. For phosphorylated glycogen synthase kinase-3 beta (p-GSK-3β[Ser9])/total GSK-3β ratio, the quantification was applied using specific ELISA kits (Cat. # 7311C for GSK-3β[Ser9] while Cat. #7265C for total GSK-3β, Cell Signaling Technology, Danvers, MA, USA). The final color reading was taken at a wavelength of 450 nm.

### 4.11. Redox Milieu

An analysis of the expression of proteins associated with SIRT1/Nrf2/HO-1 axis was conducted. The hippocampal content of SIRT1 was quantified by AFG Bioscience ELISA kit (Cat. # EK720561; AFG Bioscience, Northbrook, IL, USA). Regarding hippocampal levels of Nrf2, Cayman nuclear extraction kit was used for the isolation of total proteins in the nuclear compartment as instructed by the provider (Cat. # MBS012148; Cayman Chemical, Ann Arbor, MA, USA). Then, Nrf2 protein levels in the nuclear compartment were determined by a commercial ELISA kit (Cat. # EK720003; AFG Bioscience, Northbrook, IL, USA). The protein expression levels of HO-1 were determined by an ELISA kit purchased from Elabscience (Cat. # E-EL-R0488; Elabscience, Wuhan, China). For SIRT1, Nrf2, and HO-1, the final color reading was taken at a wavelength of 450 nm. Regarding glutathione peroxidase (GPx) activity, the enzymatic activity measurement was applied using Sigma-Aldrich GPx cellular activity kit under the guidance of the vendor (Cat. # CGP1; Sigma-Aldrich, St. Louis, MO, USA). To this end, absorbance decline at 340 nm was tracked by a kinetic program. Quantification of the lipid peroxides, a well-characterized oxidative stress marker, was performed as previously reported by Buege and Aust [54]. The final color reading was taken at a wavelength of 535 nm.

### 4.12. Statistics

Statistical analysis was conducted by the GraphPad Prism software (GraphPad Software for Science Inc., San Diego, CA, USA; version 8.0). Using Shapiro–Wilk test, we determined whether the results were normally distributed. At *p* < 0.05 as the minimal significance, Tukey–Kramer *F* test was used for statistical analysis following one-way ANOVA (parametric values). For non-parametric data, we conducted Kruskal–Wallis test followed by the multi-comparison Dunn’s test (at *p* < 0.05).

## 5. Conclusions

The current study elucidates the neuroprotective effects of linagliptin against cadmium-evoked learning and memory impairment in rats. The behavioral recovery was driven by the augmented hippocampal GLP-1 levels that lowered the neurotoxic Aβ42/p-tau and boosted acetylcholine. The associated molecular mechanisms involved combating the hippocampal oxidative insult with SIRT1/Nrf2/HO-1 pathway stimulation. Moreover, boosting hippocampal AMPK/mTOR-driven autophagy and reversal of the pro-apoptotic events were also implicated. Thus, linagliptin may be suggested as an adjunct modality for combating cadmium-induced neurotoxicity in vivo, particularly in type 2 diabetes patients with co-existing cadmium-evoked cognitive decline. Still, additional studies are needed to prove this point in the clinical setting. Moreover, a detailed description of the behavioral testing and the molecular signaling of linagliptin is demanded, including exploring several autophagy events like the expression of LC3 in the hippocampi of animals.

## Figures and Tables

**Table 1:** Study design.

Group	*n*	Received
Control	10	Animals were given normal saline as the vehicle for cadmium chloride by gavage (10 mL/kg/day). Likewise, carboxymethyl cellulose (CMC; 0.5%) was given as linagliptin vehicle by gavage to animals. Each day, the 2 doses were separated by 2 h to avoid potential interaction. The treatments were received for 8 weeks.
Control + LIN	10	Animals were given saline (10 mL/kg/day). In addition, linagliptin (5 mg/kg/day; 10 mL/kg/day) was given by gavage. Each day, the 2 doses were separated by 2 h to avoid potential interaction. The treatments were received for 8 weeks.
Cd	10	Animals were given cadmium chloride solution by gavage (5 mg/kg/day; 10 mL/kg/day). In addition, animals received an oral gavage of CMC (10 mL/kg/day). Each day, the 2 doses were separated by 2 h to avoid potential interaction. The treatments were received for 8 weeks. The chosen regimen is in accordance with published studies [29,43,44].
Cd + LIN	10	Animals were given cadmium chloride solution by gavage (5 mg/kg/day; 10 mL/kg/day). In addition, linagliptin (5 mg/kg/day; 10 mL/kg/day) was given by gavage. Each day, the 2 doses were separated by 2 h to avoid potential interaction. The treatments were received for 8 weeks. The selected linagliptin dose was based on literature that demonstrated such dose as effective for amelioration of streptozotocin-induced diabetic dementia [13] and high-fat-evoked cognitive deficit in PS19 transgenic mice [19].

## Data Availability

Data are contained within the article.
